# No effect of delivery on total hip replacement survival: a nationwide register study in Finland

**DOI:** 10.1080/17453674.2019.1628561

**Published:** 2019-06-21

**Authors:** Ilari Kuitunen, Eerik T Skyttä, Miia Artama, Heini Huhtala, Antti Eskelinen

**Affiliations:** a Faculty of Medicine and Health Technologies, Tampere University, Tampere;; b Coxa Hospital for Joint Replacement, and Faculty of Medicine and Health Technologies;; c Faculty of Social Sciences, Tampere University, Tampere;; d National Institute of Health and Welfare, Tampere, Finland

## Abstract

Background and purpose — Previous small studies have suggested that delivery does not adversely affect the survivorship of total hip replacement (THR). We investigated whether delivery after primary THR affects hip implant survivorship in a large population-based study sample

Patients and methods — In this register-based nationwide cohort study, all women aged 15–45 who underwent primary THR in Finland from 1987 to 2007 were included from the Finnish Arthroplasty Register. Data on deliveries were obtained from the medical birth register. After primary THR, 111 women (133 THRs) delivered and formed the delivery group. In the reference group, 1,878 women (2,343 THRs) had no deliveries. We used Kaplan–Meier analysis with 95% confidence intervals (CI) to study implant survivorship at 6 and 13 years, and Cox multiple regression to assess survival and hazard ratios (HRs), with revision for any reason as an endpoint with adjustment for age, rheumatoid arthritis, and stem and cup fixation.

Results — 51 (38%) revisions were recorded in the delivery group and 645 (28%) revisions in the reference group. The 6-year implant survivorship was 91% (CI 85–96) in the delivery group and 88% (CI 87–90) in the reference group. The 13-year survival rates were 50% (CI 39–62) and 61% (CI 59–64). The adjusted HR for revision after delivery was 0.7 (CI 0.4–1.2) in ≤ 6.8 years’ follow-up and 1.1 (CI 0.8–1.6) in > 6.8 years’ follow-up.

Interpretation — Based on the findings in this nationwide study of hip replacement in fertile-aged women, delivery does not seem to decrease THR implant survivorship; women should not be afraid of or avoid becoming pregnant after THR.

The most common indications for THR in very young patients aged under 30 years are rheumatoid arthritis (RA), avascular necrosis of the femoral head, and developmental dysplasia of the hip (Adelani et al. 2013). The incidence of primary THR among young patients (30 to 59 years old) has increased annually in Finland from 9.5 per 100,000 person years in 1980 to 61 per 100,000 in 2007 (Skyttä et al. 2011). In 2017, over 1,000 women aged under 55 underwent a primary THR operation in Finland (open access statistical report of the Finnish Arthroplasty Register 2018: National Institute of Health and Welfare 2018).

Only a few studies with rather small sample sizes and local data have analyzed the effects of delivery and THR on each other. None of these studies have reported problems with deliveries after THR, and they indicate that THR does not majorly affect the mode of delivery (Monaghan et al. 1987, Boot et al. 2003, Meldrum et al. 2003, Yazici et al. 2003, Sierra et al. 2005, Stea et al. 2007, Smith et al. 2008) Further, THR survival is not decreased, and the delivery method does not affect THR survival (Meldrum et al. 2003, Sierra et al. 2005). However, women have reported concerns regarding vaginal delivery and fear of delivery positions harming the THR (Ostensen 1993, Meldrum et al. 2003, Stea et al. 2007).

Very young patients seem to have worse clinical outcomes in terms of pain relief and function after THR, even though implant survival rates and radiological outcomes have improved (Adelani et al. 2013, Swarup et al. 2017). Clinical outcomes may be limited by systemic diseases, such as RA, that still comprise the majority of indications for THR in these very young patients. The survivorship of the THR is often shortened due to the loosening of cup or stem in very young patients, men, and patients with a higher BMI (Melloh et al. 2011). While in some studies underlying diseases have not negatively affected the survival of the hip prosthesis (Hannouche et al. 2016), dysplastic hips appear to have worse survival rates compared with non-dysplastic hips (Tsukanaka et al. 2016). Metal-on-metal (MoM) implants have worse survival rates compared with non-MoM implants and are since 2012 are no longer used in Finland due to common adverse local tissue reactions that have led to numerous revisions (Smith et al. 2012, Furnes et al. 2014, Varnum et al. 2015). Because THR implant survival is substantially lower in very young patients compared with older patients, THR should be considered as the treatment option of last resort for very young patients (Swarup et al. 2015, Hannouche et al. 2016).

We evaluated whether delivery adversely affects the survivorship of THR in a nationwide register-based study sample.

## Patients and methods

Data for this nationwide register-based study were gathered from 3 different national registers. Information on all women aged 15 to 45 who underwent THR operation in Finland between 1987 and 2007 was obtained from the Finnish Arthroplasty Register (FAR). The register is maintained by the National Institute for Health and Welfare (THL), and it contains information on all orthopedic prostheses operated from 1980 in Finland. All the information in the FAR has been collected prospectively. The current (2017) completeness of the register is 95% for primary THR, and it matches well with data from the Finnish Hospital Discharge Register (open access statistical report of the Finnish Arthroplasty Register 2018: NIHW 2018).

In the present study, the operation day of the primary THR was used as the starting point of the follow-up. Because we did not have information on primary THR operations before 1987, a revision THR as the first event in the FAR after January 1, 1987 was an exclusion criterion in the study. Women with bilateral prostheses were included, as earlier research has shown that this does not bias the results (Lie et al. 2004, Ranstam and Robertsson 2010). The endpoint for the follow-up was either revision, death, emigration, or December 31, 2007, whichever came first. The outcome was the revision of the hip for any reason.

**Figure 1.**
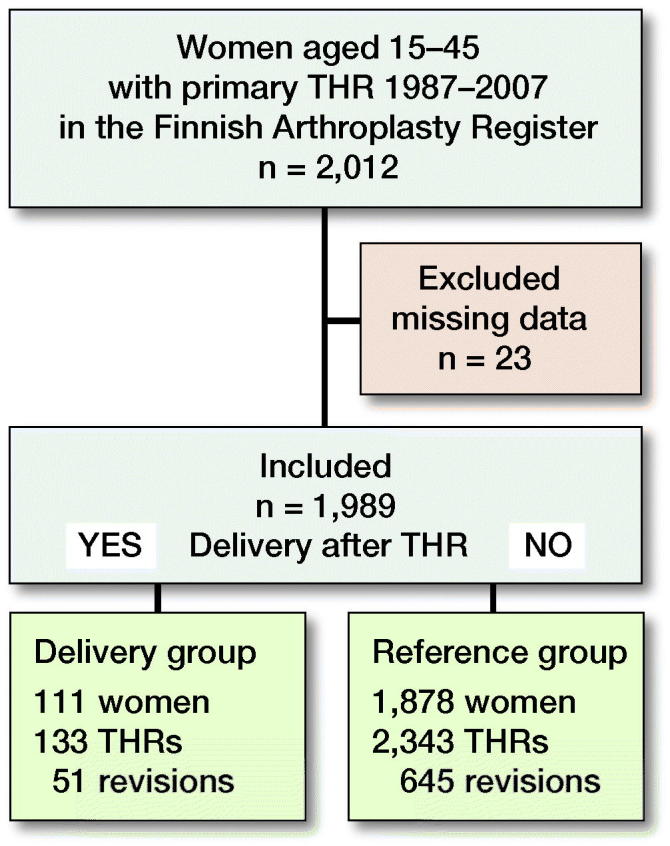
Flow chart of study population and events of total hip replacement (THR) survival among fertile-aged (15 to 45) women having delivery compared with women not having delivery after THR.

2,012 women with 2,499 primary THRs were selected from the register. Of the THRs selected, 23 were excluded due to a lack of information on many key variables (Figure 1).

Information on pregnancies and deliveries was gathered from the National Medical Birth Register (MBR) maintained by the THL. Pregnancies and deliveries from January 1, 1987 to December 31, 2007 were included in this study. The MBR contains information on all pregnancies of at least 22 gestational weeks ending in delivery and information on deliveries and newborns. MBR data match well with hospital discharge data and the coverage of the register has improved over the years. If there was no information in the MBR, the woman was not considered to have been pregnant. In the study, women who had given birth after THR formed the delivery group, and women without pregnancy after THR formed the reference group.

The Register for Reimbursable Diseases is maintained by the Finnish Social Insurance Institution of Finland. It contains information on reimbursable chronic diseases. A medical statement written by a certified doctor is needed to gain reimbursement for chronic disease. Information on all the reimbursements for this study population was obtained. If there was no information available, women were considered as not having chronic diseases.

In this study, RA was the most common diagnosis. Other chronic diseases were rare, but the following diseases were found: asthma, diabetes mellitus type 1, epilepsy, hypothyroidism, hypertension, inflammatory bowel disease, and major psychiatric disease.

### Statistics

Categorized variables were compared by chi-square test between the groups and reported as proportions. Continuous variables were compared by their distribution. Normally distributed variables were compared by Student’s t-test and reported by means with standard deviations (SD). Non-normally distributed variables were compared by Mann–Whitney U-test and reported by medians with interquartile range. A p-value under 0.05 was considered statistically significant in all analyses. Kaplan-Meier survival analyses with 95% confidence intervals (CI) were performed to evaluate the survival of the hips in both the delivery group and the reference group. Survival rates were calculated for 6 years’ and 13 years’ follow-up. The follow-up was continued until 13 years when 20 THRs were still at risk (life table analysis) in the delivery group. The follow-up period was calculated from primary THR until revision THR or until the date the patient was censored at the end of the study (December 31, 2007), or date of emigration, or date of death. The Cox proportional hazards model was used to analyze the effect of potential confounders and count hazard ratios (HR). The adjustments used in the Cox proportional analysis were the following: age at the time of primary THR, RA, stem fixation, and cup fixation. Because the proportional hazards assumption was not met in the Cox model (crossing survival curves at 6.8 years), the follow-up was divided into 2 time periods, and a piecewise Cox proportional model was performed. The first follow-up period was the time before the crossing at 6.8 years, and the second period was from the crossing until the end of the follow-up (6.8–21.0 years). All the analyses were performed using SPSS statistical software version 25.0 (IBM Corp, Armonk, NY, USA).

### Ethics, registration, funding, and potential conflicts of interest

In accordance with Finnish regulations, informed patient consent was not required as the women were not contacted. Our study protocol went through the ethical evaluation of the National Institute for Health and Welfare to gain access to register data, permission number: THL/599/5.05.00/2010.This study was funded by the Competitive Research funds of Pirkanmaa Hospital District, Tampere, Finland, representing governmental funding. The authors have no potential conflicts of interests to declare.

## Results

1,989 women with 2,476 THRs were included in the study (Table 1). Of these, 111 (5.6%) women with 133 (5.4%) THRs had a delivery during the follow-up. The mean follow-up in the delivery group was 9.3 years (0–21), and the median age at the start of the follow-up was 29 years. In the reference group, 1,878 women with 2,343 THRs had no deliveries. The mean follow-up was 8.1 years (0–21), and the median age at the start of the follow-up was 40.

**Table 1.** Background characteristics of the study population, types of hip prosthesis, and indications for revisions between the delivery group and the reference group. Values are frequency (%) unless otherwise specified

Factor	Delivery group n = 133	Reference group n = 2,343
Age at primary THR^a^	29 (8)	40 (8)
Follow-up period (years)^a^	9.1 (6)	8.0 (8)
Rheumatoid arthritis	62 (47)	774 (33)
Other chronic disease^b^	5 (4)	208 (9)
Nulliparous at primary THR	78 (64)	778 (33)
Indication for THR		
Inflammatory arthritis (RA + others)	62 (47)	731 (31)
Primary osteoarthritis	12 (9)	532 (23)
Secondary arthrosis	21 (16)	363 (16)
DDH^c^	22 (17)	493 (21)
Other	16 (12)	224 (10)
Metal-on-metal bearing	16 (12)	390 (17)
Type of primary THR fixation		
Uncemented	114 (86)	1,859 (79)
Hybrid	7 (5)	237 (10)
Inverse hybrid	0 (0)	1 (0)
Cemented	12 (9)	245 (10)
Revisions	51 (38)	645 (28)
Revision indications		
Aseptic loosening	30 (59)	318 (50)
Deep infection	1 (2)	11 (2)
Periprosthetic fracture	0 (0)	12 (2)
Dislocation	1 (2)	30 (5)
Others	14 (27)	193 (30)
Missing	5 (10)	81 (12)

a Median and interquartiles.

b Includes: asthma, diabetes mellitus type 1, epilepsy, hypothyroidism, hypertension, inflammatory bowel disease, major psychiatric disease.

c DDH = developmental dysplasia of the hip.

RA was the most common indication for THR in both groups. It was, however, more prevalent in the delivery group (47%) than in the reference group (33%) (p = 0.001). Other chronic diseases were more common in the reference group. The distribution of THR fixation method or bearing type was similar between the groups. The delivery group had 51 revisions, and 30 (59%) of the revisions were performed due to aseptic loosening. In the reference group, 645 THRs were revised, and 318 (49%) revisions were performed due to aseptic loosening.

The deliveries were analyzed and recorded per THR. 170 deliveries occurred during the follow-up (mean of 1.3 deliveries per THR). The maximum number of deliveries per patient during the follow-up was 5. Of the deliveries, 75 (44%) were vaginal and 95 (56%) Cesarean sections. 50 women with 53 THRs had at least 1 vaginal delivery after THR and 61 women with 80 THRs had only Cesarean sections after THR. The primary THR diagnoses and revision indications were similar in the vaginal delivery group and Cesarean section group (Table 2).

**Table 2.** Comparison of primary diagnoses and revision indications in the delivery group between women with at least 1 vaginal delivery after total hip replacement (THR) with women with only Cesarean sections after THR

Factor	Vaginal delivery after THR n = 53	Cesarean section after THR n = 80
Indication for THR		
Inflammatory arthritis (RA + others)	19	43
Primary osteoarthritis	4	8
Secondary arthrosis	13	8
DDH	10	12
Other	7	9
Revisions	15	36
Revision indications:		
Aseptic loosening	10	20
Deep infection	1	0
Dislocation	0	1
Others	4	10
Missing	0	5

DDH = developmental dysplasia of the hip.

**Figure 2.**
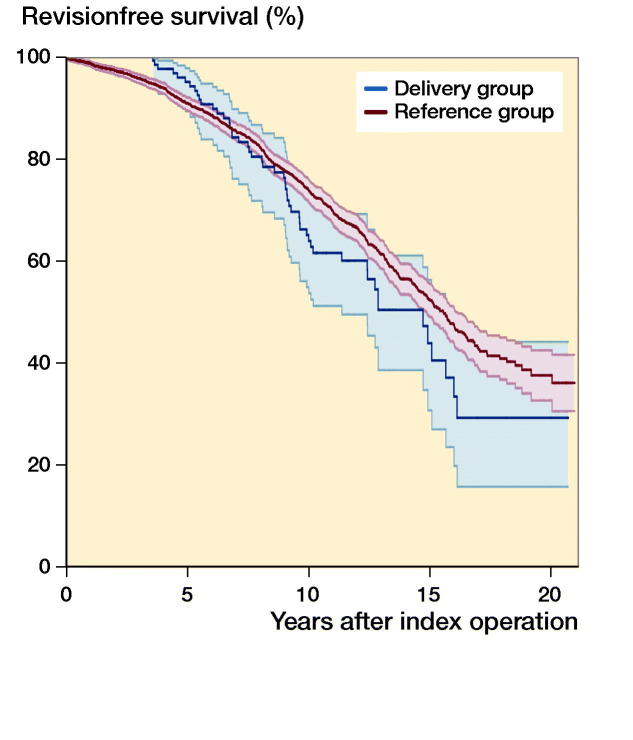
Kaplan–Meier survival curves (with 95% confidence intervals) of primary total hip replacement (THR) among fertile-aged women aged 15 to 45 years at the time of THR having 1 or more deliveries after THR (delivery group) compared with no deliveries after THR (reference group).

At 6 years, the survival rate in the delivery group was 91% (CI 85–96) and in the reference group 88% (CI 87–90). At 13 years, the survival rate was 50% (CI 39–62) for the delivery group and 61% (CI 59–64) for the reference group, respectively (Figure 2, Table 3).

**Table 3.** Kaplan–Meier 6- and 13-year survival rates with 95% confidence intervals (CI) of primary total hip replacement of fertile-aged women aged 15 to 45 years at the time of THR

Delivery	No. of hips	No. of revisions	K–M survivorship at 6 years		at 13 years	
			No. at risk	survival % (CI)	No. at risk	survival % (CI)
Yes	133	51	100	91 (85–96)	22	50 (39–62)
No	2,343	645	1,411	88 (87–90)	456	62 (59–64)

During the first time period (0 to 6.8 years’ follow-up), the adjusted Cox regression model showed no statistically significant difference in the risk for revision between the delivery and the reference groups (adjusted HR 0.7, CI 0.4–1.2). During the later follow-up (6.8 to 21 years), there was still no difference in adjusted HR between the groups (HR 1.1, CI 0.8–1.6).

## Discussion

To our knowledge, our study is the first to assess THR implant survivorship in fertile-aged women in a large population-based study sample. Based on our results, delivery does not seem to adversely affect hip implant survivorship after primary THR.

Our results are in concordance with previous smaller studies. In their study, Sierra et al. (2005) reported that delivery after primary THR does not decrease the survival rate of the implant. They had the largest number of participants prior to our study. 343 women with 420 THR were contacted and 47 of those had pregnancy ending in delivery. However, the survival rates for 5-, 10- and 15-year follow-up periods were calculated for the whole cohort with no comparisons made between the delivery and non-delivery groups. Our 6-year survival rate in both groups was in line with these results. Meldrum et al. (2003) had 13 hips with deliveries in their study population and reported no adverse effects for THR. Yazici et al. (2003) reported 21 THR patients with deliveries and no decrease in the survival rate of the THR. All these studies were retrospective with alternative response rates (30–75%). McDowell and Lachiewicz (2001) reported 5 women with 7 uncemented THRs having deliveries and, compared with matched referents, no differences between survival or hip functions were reported. Our study was the only one to report a slight but not statistically significant decrease in implant survival rate in the Kaplan–Meier analysis after delivery.

Cesarean section (CS) rate was markedly increased in the delivery group compared with overall CS rate in Finland. There have been previous reports in which women with dysplastic hips have been discussed to have smaller pelvic diameters and therefore could tend to have CS (Sierra et al. 2005; Stea et al. 2007). Developmental dysplasia of the hip was an equally common indication for THR in women who only had Cesarean sections after THR as in those who delivered vaginally after THR in our study. Also, revision indications did not differ between them. The reason for the very high CS rate in the delivery group remains unknown. We can only speculate that the presence of THR may have affected the patients’ and/or the physicians’ choice of delivery. However, it did not have any effect on THR survival rates.

Age was the only statistically significant variable that negatively affected THR implant survivorship. The delivery group’s median age at the start of the follow-up was 29 years compared with the reference group’s 40 years. Previously, only Sierra et al. (2005) have applied the Cox regression model to analyze implant survivorship after delivery. In their model, delivery seemed to decrease THR survivorship, but once age at the time of primary THR was taken as part of the model, no further differences between the delivery group and the reference group were obtained. Previous non-delivery-related THR survival studies have reported similar findings of weaker implant survivorship in younger patients (Dorr et al. 1994, Nam et al. 2016, Tsukanaka et al. 2016). In particular, very young patients under 30 years have been reported to have had decreased THR survivorship (Mohaddes et al. 2019) probably because of higher activity levels (Dorr et al. 1994, Adelani et al. 2013). Our survival rates were slightly lower compared with the recent study of Mohaddes et al. (2019), in which the 15-year THR survival rate for patients aged under 30 at the time of THR was 76%.

In young patients (< 50 years or less), indications for THR differ in comparison with older patients (+50 years). In younger patients, inflammatory arthritis and developmental hip diseases are more common, and primary osteoarthritis is rare (Adelani et al. 2013). Developmental dysplasia of the hip decreases the survival of the hip prosthesis in young patients (Havelin et al. 2000, Tsukanaka et al. 2016). There have been controversial results regarding the survival of the THR in RA patients. Some studies have suggested decreased THR survivorship, more common radiographic findings indicating implant failure, poorer function, and increased mortality among patients with RA (Creighton et al. 1998, Havelin et al. 2000, Tang and Chiu 2001, Singh and Lewallen 2013, Goodman et al. 2014, Schrama et al. 2015). Inflammatory arthritis as primary diagnosis for THR may also increase revisions due to deep infections (Dale et al. 2012). Previous large national cohort studies, however, have shown no decrease in THR survival due to RA (Havelin et al. 2000, Furnes et al. 2001, Eskelinen et al. 2006). Because of the high prevalence of RA among young patients, it was taken as part of the Cox model. In our model, RA did not decrease THR survival. Indeed, it seemed patients with RA had better results during the first follow-up period (< 6.8 years). A similar finding was seen in a previous THR and delivery study, where Serra et al. (2005) found no decrease in the survival of hips operated due to an RA diagnosis in their step-by-step Cox results.

The main strength of our study is the register-based design. Previous THR survival studies after delivery have been retrospective cohorts with questionnaires. Our design eliminates possible recall bias and has better completeness because revision indications are also reported to the Finnish Arthroplasty Register. In addition, we had by far the largest study population with the longest follow-up, and our results are nationwide instead of from one hospital district catchment area. In addition, we were also able to combine information from several nationwide registers on patients’ long-term diseases and pregnancies.

The first limitation of our study is the lack of patient-reported outcome measurements (PROMs), which forced our study to focus strictly on the survival of the implant. However, absence of PROM data does not affect our interpretation of the survival results. The second limitation was the study period. Our study period was from 1987 to 2007. Even though the implants used today differ greatly from those implanted 30 years ago, contemporary implant designs were used in the latter half of the study period, and this approach also enabled us to assess long-term implant survivorship in this rare cohort of patients.

In conclusion, based on the findings in this nationwide study offering hip replacement to fertile females, delivery does not seem to decrease THR implant survivorship. Women should not be afraid of or avoid becoming pregnant after THR.
